# Anaplastic Lymphoma Kinase‐Rearranged Chest Wall‐Undifferentiated Small Round Cell Sarcoma With Massive Pleural Effusion and Rapid Progression: A Case With Autopsy Report

**DOI:** 10.1111/1759-7714.70160

**Published:** 2025-09-04

**Authors:** Toshiki Amioka, Kaori Okayasu, Shoko Iwanaga, Mio Yamamoto, Mizuho Tosaka, Toshihisa Ishikawa, Tsutomu Kawasaki, Takehiko Shimoyama, Jiro Kumagai

**Affiliations:** ^1^ Yokohama City Minato Red Cross Hospital, Respiratory Medicine Yokohama Kanagawa Japan; ^2^ Hiratsuka Kyosai Hospital, Respiratory Medicine Hiratsuka Kanagawa Japan; ^3^ Yokohama City Minato Red Cross Hospital, Thoracic Surgery Yokohama Kanagawa Japan; ^4^ Yokohama City Minato Red Cross Hospital, Pathology Yokohama Kanagawa Japan

**Keywords:** chest wall, EML4‐ALK, pleural effusion, small round cell sarcoma

## Abstract

Chest wall sarcomas are rare and may exhibit aggressive behavior, posing diagnostic challenges—particularly in young adults. Although multidisciplinary treatments involving chemotherapy, radiotherapy, and surgery are recommended, prognosis remains poor. We report a case of a 43‐year‐old man referred with left‐sided chest pain, dyspnea, and massive pleural effusion. Cytological analysis of the effusion and biopsy revealed small, round, atypical cells, and initial immunohistochemistry suggested Ewing sarcoma. During workup, the patient's symptoms worsened, tumor lysis syndrome developed, and he died on hospital day 28. Autopsy and extended immunohistochemical testing indicated small round cell sarcoma. Molecular analysis identified an echinoderm microtubule‐associated protein‐like 4 (*EML4*)‐anaplastic lymphoma kinase (*ALK*) fusion gene. The final diagnosis was small round cell sarcoma with EML4‐ALK fusion originating from the thoracic wall. This case highlights the importance of early presentation and timely diagnosis using next generation sequencing to facilitate targeted therapy for *ALK*‐rearranged chest wall sarcomas and improve patient outcomes.

## Graphical abstract

Early molecular diagnosis using NGS may facilitate targeted therapy and improve outcomes in rare chest wall sarcomas.

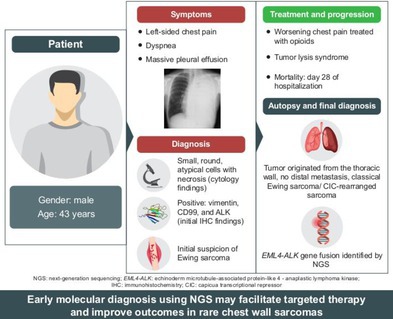

## 1 Introduction

Chest wall sarcomas are rare, sometimes aggressive, and often difficult to diagnose, particularly in young adults. They represent a heterogeneous group of tumors with diverse origins, clinical behavior, histology, and outcomes [1]. Although multidisciplinary treatment—including chemotherapy, radiation therapy, and surgery—is recommended, prognosis remains uncertain. We describe a man with rapidly progressive primary chest wall sarcoma, initially resembling Ewing sarcoma histologically. Subsequent testing identified an echinoderm microtubule‐associated protein‐like 4 (EML4)‐anaplastic lymphoma kinase (ALK) gene fusion, with no other notable mutations. This rare case underscores the importance of timely, multidisciplinary diagnosis to guide appropriate treatment.

## 2 Case Report

**FIGURE 1:**
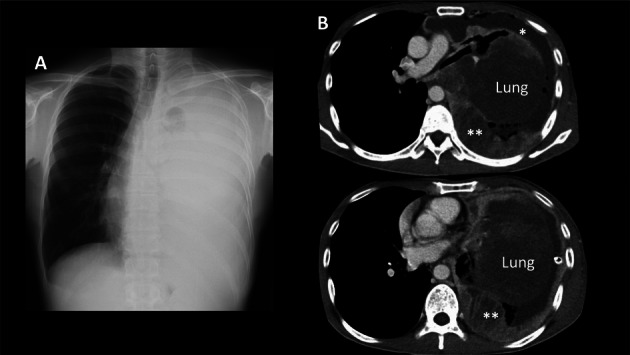
Chest X‐ray (A) and enhanced CT (B) at the time of the diagnosis, revealing massive left pleural effusion (*) and compressed lung, resulting in a rightward shift of the mediastinum. The left pleura was irregularly thickened on the left side of the back (**). Enlarged lymph nodes or distal metastases were not observed. CT, computed tomography.

**FIGURE 2:**
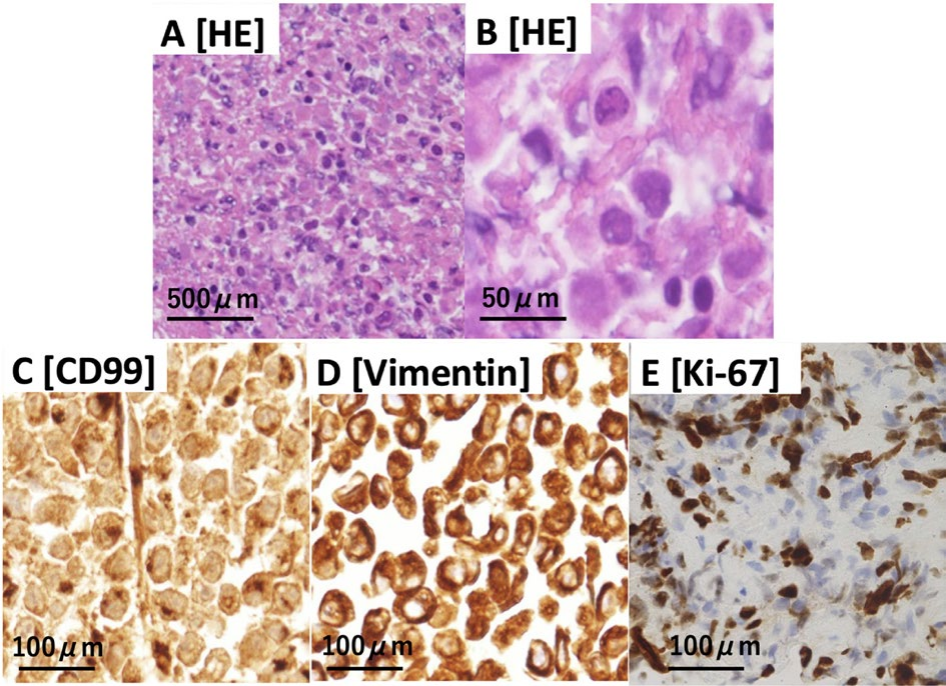
HE staining of the pleural fluid cell block reveals numerous necrotic cells and small round cells (A, B). Immunohistochemical analysis showing positivity for CD99 (C) and vimentin (D), with a markedly high Ki‐67 index (E). CD99, cluster of differentiation 99; HE, hematoxylin and eosin.

A 43‐year‐old man, a non‐smoker with no history of dust exposure, presented with progressive left‐sided chest pain, several months of dyspnea, and massive pleural effusion. He had untreated atopic dermatitis and showed low‐grade fever, tachycardia, but no tachypnea. Oxygen saturation was 95% and auscultation revealed decreased breath sounds on the left. Chest radiography and contrast‐enhanced CT showed left lung atelectasis and rightward mediastinal shift due to the effusion (Figure 1). Irregular posterior pleural thickening was noted on the left, without lymphadenopathy or distant metastases. Laboratory tests indicated inflammation: white blood cell count 20,500/μL, eosinophils 17.9%, C‐reactive protein 6.7 mg/dL, and lactate dehydrogenase level 416 U/L. Among tumor markers, only soluble interleukin‐2 receptor was elevated (1737 U/mL). Diagnostic thoracentesis yielded hemorrhagic, exudative effusion with atypical cells. A pleural fluid cell block revealed small, round, poorly differentiated tumor cells with necrosis and structural disarray. Immunohistochemistry was positive for CD99 and vimentin, negative for TTF‐1, P‐40, and CD56; Ki‐67 was positive in 50% to 60% of cells (Figure 2). Medical thoracoscopy revealed irregular, edematous pleural thickening. Biopsy confirmed small, round, undifferentiated tumor cells with necrosis, consistent with a mesenchymal rather than epithelial origin.

**FIGURE 3:**
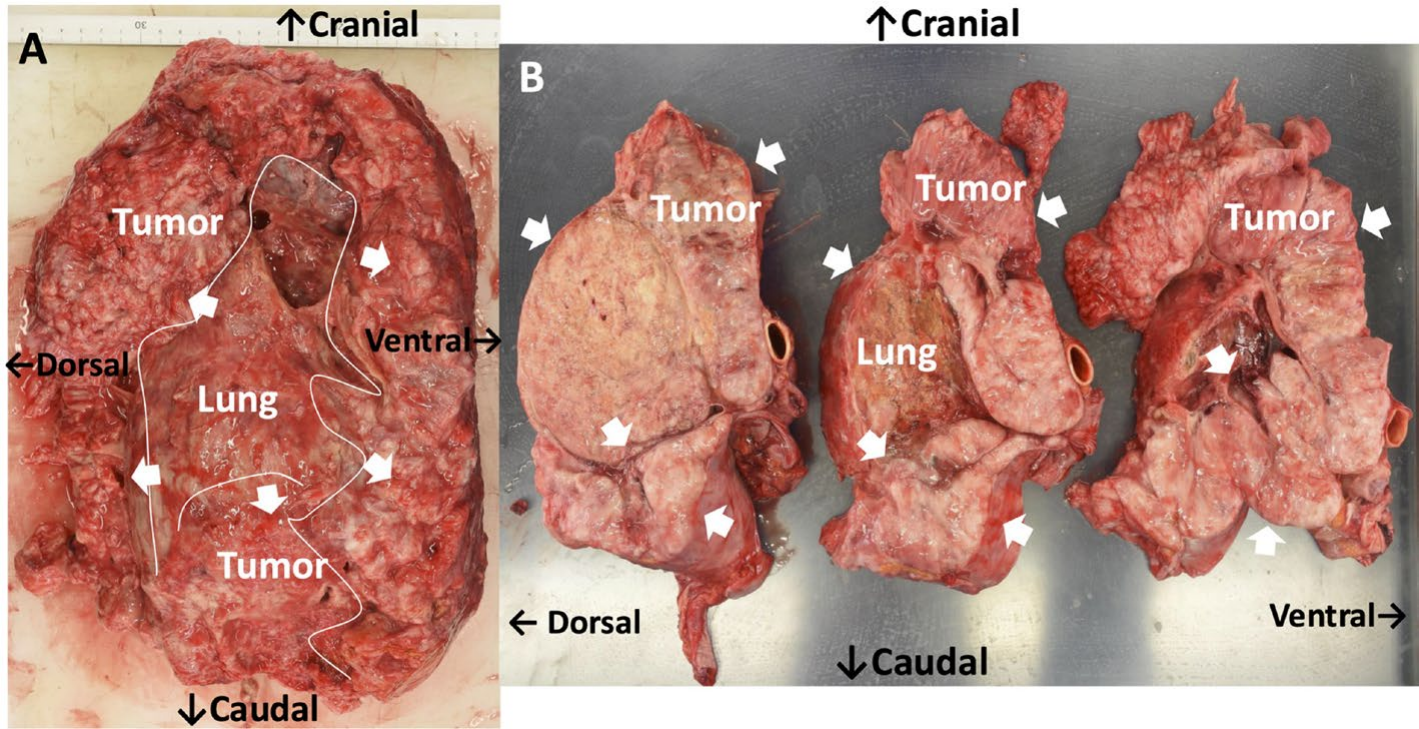
Macroscopic findings from an autopsy. White arrows show the tumor occupying the left cavity from the thoracic wall. (A) A full image of the left large tumor and left lung. A large tumor occupies the left thoracic cavity, covering the front, side, and apical chest walls and invading the front intercostal muscle. (B) Sagittal planes reveal a thick tumor originating from the thoracic wall, and the cut surface of the tumor reveals yellowish‐white necrosis.

**FIGURE 4:**
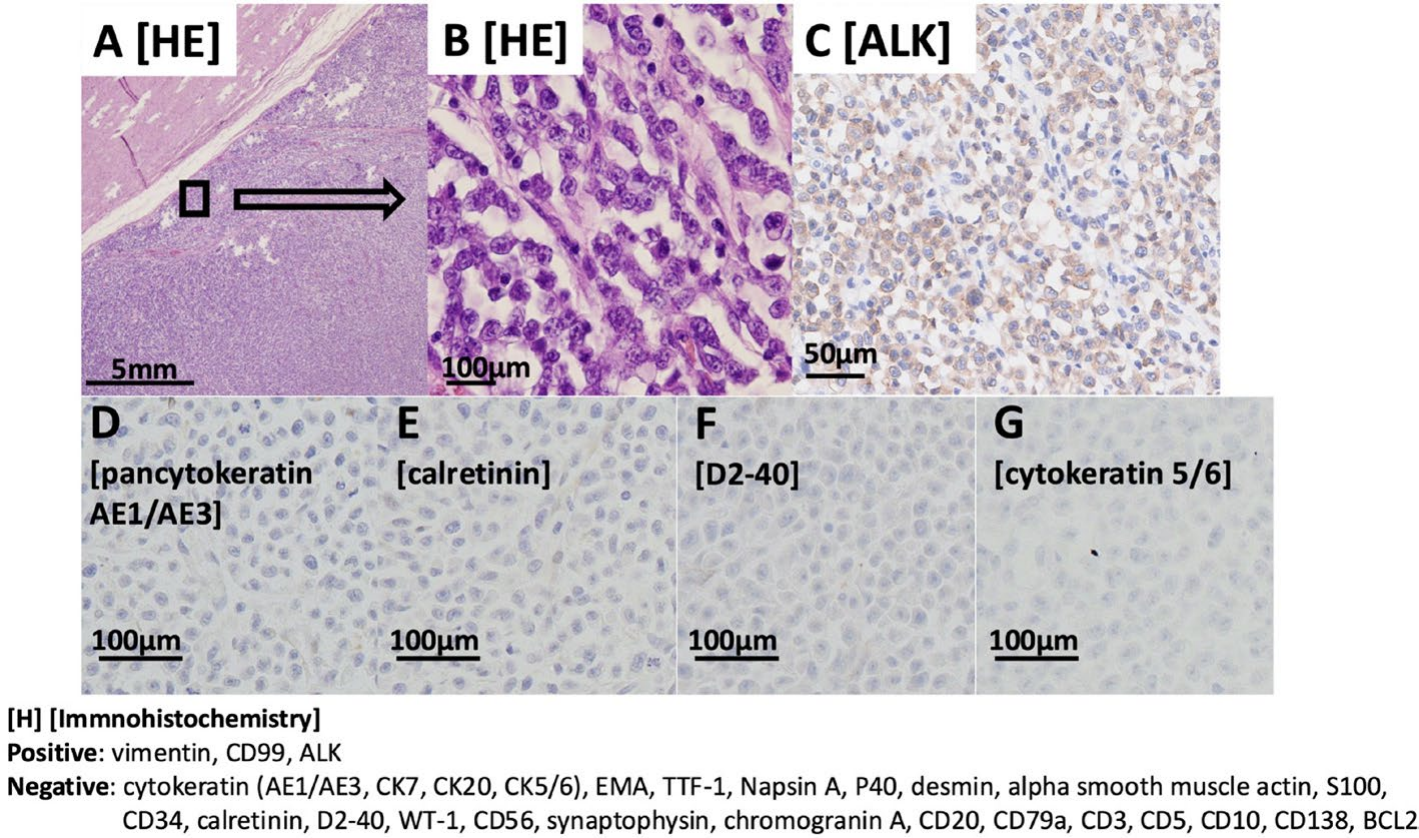
Histological findings from the autopsy showing no invasion of the aorta (A), and showing small round cells of a high nuclear‐cytoplasmic ratio lacking their epithelial structure (B). Immunostaining for anti‐ALK antibody was positive (clone: 5A4) (Leica) (C). Immunostaining for pancytokeratin AE1/AE3, calretinin, D2–40, and cytokeratin5/6 was negative (D–G). Detailed results of immunohistochemical staining are presented (H). ALK, anaplastic lymphoma kinase; BCL2, B‐cell lymphoma 2; CD34, cluster of differentiation 34; CD99, cluster of differentiation 99; CK7, cytokeratin 7; EMA, epithelial membrane antigen; HE, hematoxylin and eosin; TTF‐1, thyroid transcription factor‐1; WT‐1, Wilms tumor 1.

During the diagnostic period, the patient required opioids for worsening left‐sided chest pain, and tumor lysis syndrome progressed. Due to poor performance status, he was ineligible for multimodal therapy, including chemotherapy and palliative radiotherapy, and was transitioned to best supportive care. He died on hospital day 28. Autopsy revealed a large left thoracic tumor occupying the anterior, lateral, and apical chest walls (Figure 3A). It invaded the anterior intercostal muscles and adhered to—but did not infiltrate—the pericardium or descending aorta. Sagittal sections showed a thick, necrotic, yellowish‐white tumor arising from the thoracic wall (Figure 3B). No distal metastases were identified. Histopathology showed small round tumor cells with high nuclear‐to‐cytoplasmic ratios, brisk mitosis, and no epithelioid features (Figure 4B). There was no invasion of the esophagus or aorta (Figure 4A). These findings indicated a thoracic wall origin rather than pulmonary. Additional immunohistochemistry (Figure 4C–H) excluded epithelial tumors, lung cancer, mesothelioma, neuroendocrine tumors, and lymphoma. A diagnosis of small round cell sarcoma was made. Whole RNA sequencing (RIKEN Genesis Co. Ltd., Tokyo, Japan) ruled out Ewing sarcoma and Capicua transcriptional repressor (CIC)‐rearranged sarcoma but detected an *EML4* exon 14‐*ALK* exon 20 fusion. Based on these findings, we diagnosed small round cell sarcoma with *EML4*‐*ALK* fusion originating from the thoracic wall.

## 3 Discussion

This report describes a young man with a rapidly progressive undifferentiated small round cell sarcoma of the thoracic wall harboring an *EML4*‐*ALK* gene fusion. Unfortunately, diagnostic challenges and rapid disease progression precluded the initiation of multimodal anticancer therapy. A definitive diagnosis of sarcoma was supported by detailed postmortem immunohistochemical analysis.

Anaplastic small round cell sarcomas of the bone and soft tissue are high‐grade, aggressive tumors with poor prognoses, particularly in children and young adults. Based on immunohistochemical results, both Ewing sarcoma and CIC‐rearranged sarcoma were considered in this case. Recent advances in genomic profiling have enabled the identification of characteristic genetic alterations, and these have been incorporated into the updated 2020 WHO classification of the soft tissue and bone tumors [2]. However, molecular analysis did not detect gene fusions typical of Ewing sarcoma or CIC‐rearranged sarcoma; instead, an *EML4*‐*ALK* fusion was identified postmortem. The EML4‐ALK fusion is the most common *ALK* rearrangement in non‐small cell lung cancer, and multiple ALK tyrosine kinase inhibitors have been approved [3]. *ALK* rearrangements are also observed in inflammatory myofibroblastic tumors [4]. Additionally, although rare, several reports describe successful ALK‐targeted therapy in sarcomas with ALK fusions [5, 6, 7]. In one study, three of seven patients with ALK‐positive mesenchymal tumors responded to ALK inhibitors [8].

In this case, diagnostic delays, progressive chest pain, and the development of tumor lysis syndrome prevented treatment initiation. This case highlights the importance of early referral and rapid diagnostic workup, including next generation sequencing, to facilitate effective gene‐targeted treatment and improve outcomes and quality of life.

## Author Contributions

T.A.: Writing – original draft. K.O., S.I., M.Y., M.T., T.I., T.K., T.S., and J.K.: Writing – review and editing. K.O.: Conceptualization. All the authors approved the final version of the manuscript.

## Disclosure

The authors have nothing to report.

## Ethics Statement

Informed consent was obtained from the family of the patient.

## Consent

The family of the patient provided informed consent for the publication of this report.

## Conflicts of Interest

The authors declare no conflicts of interest.

## Data Availability

The data that support the findings of this study are available from the corresponding author upon reasonable request.
